# The Association Between Demographic Characteristics and Attempting of Pregnancy with Postpartum Depression and Anxiety Among Women Referring to Community Health Centres: A Cross Sectional Study

**DOI:** 10.21315/mjms2020.27.3.10

**Published:** 2020-06-30

**Authors:** Maryam Alikamali, Sedigheh Khodabandeh, Maryam Motesaddi, Zeinab Bagheri, Mohammad Ali Esmaeili

**Affiliations:** 1 Student Research Committee, Kerman University of Medical Sciences, Kerman, Iran; 2 Nursing Research Center, Kerman University of Medical Sciences, Kerman, Iran

**Keywords:** anxiety, depression, pregnancy, attempting of pregnancy, social support

## Abstract

**Background:**

Postpartum depression (PPD) and anxiety are considered as a risk factor for mother and infant health. Therefore, the present study aims to explore the association between demographic characteristics and pregnancies with PPD and anxiety.

**Methods:**

A cross-sectional study was conducted on 400 Iranian women referring to health centres of the Zarand City four weeks to six months from the date of their childbirth, in the first half of 2018.

**Result:**

The results showed that employed women with pregnancies who were categorised as depression and anxiety were more likely to have low gestational age, food insecurity, several deliveries, cesarean delivery and unintended pregnancy as well as they were not satisfied with their infant’s gender. Also, women with several deliveries had lower risk for PPD before and after adjustment for confounders (odds ratio [OR] = 0.92, 95% confidence interval [CI]: 0.88–0.97, *P* < 0.001) and had lower risk for postpartum anxiety only after adjustment for confounders (OR = 0.82, 95% CI: 0.75–0.89, *P* < 0.001).

**Conclusion:**

Eventually, demographic characteristics and attempting of pregnancy were independently associated with PPD and postpartum anxiety in women. There need to be more social and governmental support of employed women after delivery to decrease their occupational stresses to deal with PPD and anxiety in the studied population.

## Introduction

Postpartum depression (PPD), as major depression, is a complex mix of physical, emotional, and behavioural changes that happen in a woman after a childbirth. Mental and physical disorders caused by PPD can create problems for the mother, infant and other family members, and may affect the level of the mother-infant bonding and other family relationships (1). Research shows that PPD is a silent killer that causes mothers mortality and can have unpleasant consequences for the health and growth of the infants (2). While European, Australian and United States of America (USA) women appear to have lower levels of PPD, women from Asia and South Africa have been identified as being most at PPD risk (3). It has been reported that prevalence rates of depression in postpartum women are between 3.5%–63.3% in Asian countries (4, 5).

Also during pregnancy, anxiety, as a factor responsible for negative maternal and child outcomes is one of the most common symptoms (6). Anxiety prevalence during pregnancy, in developed and developing countries is 10% and 25%, respectively (7, 8). In the population of Iranian pregnant women, the prevalence of anxiety has been reported by 15% (9). It has been consistently found that Antenatal anxiety is a strong predictor of postnatal anxiety and depression (10).

According to the results of Ganjouei et al. (11), there is a significant correlation between depression and economic status, type of childbirth, unintended pregnancy, history of depression in pregnancy and problems with the husband’s family (11).

Unintended pregnancy, as a considerable factor, is one of the most common problems in the third world and developing countries and it is a cultural-health problem that affects women, families and society. The unintended pregnancy is generally described as a pregnancy that occurs in an inappropriate time (mistimed), when one of couples or both of them are unwilling to have children (12, 13). The prevalence of unwanted pregnancy in Iran is about 27% (14, 15). Although there are many methods for preventing pregnancy, however, unintended pregnancy occurs due to the lack of use or inappropriate use of these preventive methods (16). According to the World Health Organization (WHO) in 2018, from 214 million pregnancies per year, 85 million of them were unintended pregnancy (11). Due to the lack of family support, unintended pregnancy is considered as a risk factor for the health of the mother and their infant. Unintended pregnancy has harmful effects on the mother and infant and generally on the family, including abortion, inadequate prenatal care, reducing the quality of mother-infant bonding and the child’s emotional, physical abuse and violence against women, etc. (11, 17–19). Based on the results of Khajehpour et al. (20), women with unintended pregnancies compared with women with intended pregnancy gained lower scores for physical and mental status for self-care behaviours, such as the use of supplements and caring herself during pregnancy (21). However, exposure to unplanned pregnancy and disagreement with it brings pregnancy with more fear and more anxiety (22–24). To a large extent, factors such as suicide, drinking alcohol and smoking have a great impact on unwanted pregnancies (9). Reducing the unwanted pregnancy rate is necessary in order to increase positive health outcomes (25).

Therefore, the aim of this study was to investigate the association between demographic characteristics and attempting of pregnancy with PPD and anxiety and depression among women referring to community health centres.

## Methods

### Study Design and Setting

This research is a cross-sectional study conducted in an urban area of Zarand City, Iran from January 2018 to June 2018. Before starting the experiment, the women were informed of the study purpose and process. Those women who expressed an interest to participate in this study signed the consent form for a research questionnaire.

### Study Participants, Sample Size and Sampling Strategy

This cross-sectional study was conducted on 400 Iranian mothers referred to health centres in the Zarand City for four weeks to six months after delivery. The study population was collected from all the regions of Central and West Zarand, using community-based sampling based on cluster sampling. The sample size in this study was calculated around 400 women using the GPOWER software. A subset of 683 women was offered enrollment in the study after their deliveries. Among 683 mothers, 400 mothers were included and 283 subjects left, according to the inclusion and exclusion criteria for the current analysis. The eligibility criteria to participate in the study included Iranian nationality and willingness to participate in the study. Complicated pregnancy (having diabetes, hypertension, pulmonary, heart and kidney diseases, and anemia during pregnancy) and taking psychiatric medication were the criteria excluded from the study. All cases signed consent forms to take part in the study. The survey tool was the demographic information which include age, educational level, occupation, spouse’s job, income, numbers of childbirth, type of delivery (vaginal versus cesarean) and satisfaction with the newborn’s gender.

Pregnancy intention was assessed with a modified version of the Centres for Disease Control and Prevention (CDC)’s Pregnancy Risk Assessment and Monitoring System (PRAMS) questions (26). Women who were attempting pregnancy at the time of conception and women who were not attempting pregnancy at the time of conception, but who had intended to become pregnant at some future time were defined as having an ‘intended’ pregnancy. Women who were not attempting pregnancy at the time of conception, and stated they had not intended a pregnancy at any future time were defined as ‘unintended’ pregnancy.

The food security status of the households was assessed by a food security questionnaire submitted by the US Department of Agriculture (USDA). This 18-item questionnaire examines household food security status over the past 12 months, was completed by interviewing mothers. The USDA 18-item questionnaire has recently been validated in Isfahan City families in Iran (27).

Edinburgh postpartum depression (EPDS) inventory was used (28), which includes 10 questions with four answers for each question with a score of 0 to 3, based on the type of person’s response. The score of the cutoff point of the study was 12 and those who scored 12 or higher were considered as the depressed people and subjects with a score under 12 as the normal people.

The Spielberger State-Trait Anxiety Inventory (STAI), which is a self-report tool, consists of 40 questions that 20 questions are related to apparent anxiety and 20 other questions were devoted to hidden anxiety and 4-points Likert scales were used by a total score of 20 to 80 for each section. The types of anxiety based on the test scores were classified: mild anxiety (20–39 points), moderate anxiety (40–59 points) and severe anxiety (60–80 points) (29). The Cronbach’s alpha was obtained by a pre-test of a 30-member sample and it was 0.86 for the depression inventory and 0.84 for the anxiety inventory. To collect information, the researchers after coordinating with the health centre authority went to the midwifery section of the determined health centres in and explained the study objectives for the mothers and those who were willing to participate in the study were selected. The confidentiality of the gathered information and its use in the study was emphasised for the subjects. Then the inventory was completed by the researcher and interviewing with the research units.

Data analysis was performed using SPSS version 19.0 software (SPSS Inc., Chicago, IL, USA). We examined bivariate associations using the Chi-square test. First, frequency distributions of the characteristics of the study population were tabulated. Next, bivariate analysis was done to compare depressive and anxiety symptoms by study characteristics using Chi-square tests. We categorised anxiety in two levels for analysis, moderate and severe anxiety categorised in the same level and mild anxiety.

Binary logistic regression was utilised to study unadjusted and adjusted associations between PPD and postpartum anxiety, as well as other demographic and characteristics of mothers in the sample and to estimate odds ratios (ORs) and 95% confidence intervals (CIs) for the various categories. Hosmer and Lemeshow test was used to assess the goodness of fit (30). The *P*-value less than 0.05 (*P* < 0.05) was considered statistically significant.

## Results

Descriptive statistics included in examining frequency distributions of the categorical variables and assessing for missing data. Among 400 women who completed the questionnaires, none of 400 subjects dropped out of the study before completion.

Table 1 describes the demographic characteristics of mothers included in the samples. Of these, 65% had pregnancies categorised as intended and 35% had pregnancies categorised as unintended. Most of them belonged to a gestational age of more than 37 weeks (363 women, 90.7%) and 208 of these women were housewives (52.5%), and the rest of them had a job. The vaginal delivery was 56.2% (225 women) and the cesarean delivery was 43.5% (175 women). Mothers who were satisfied with their newborns’ gender were 260 (65%) people and 140 (35%) of them were not satisfied with their infant’s gender.

We examined bivariate associations using the Chi-square test for the relationship between gestational age, food’s status, demographic characteristics, type of pregnancy, delivery type, satisfaction with the gender and PPD and anxiety in attempting of pregnancy pregnancies. Some characteristics showed a relationship of statistically significant with PPD and postpartum anxiety. Women with pregnancies categorised as depression were more likely to have low gestational age (*P* < 0.001), food insecurity (*P* = 0.039), several deliveries (*P* = 0.004), high total household income (*P* = 0.008), cesarean delivery (*P* = 0.026) and were not satisfied for their infant’s gender (*P* = 0.002). Also, women with pregnancies categorised as anxiety were more likely to have low gestational age (*P* < 0.001), food insecurity (*P* = 0.048), unintended pregnancy (*P* = 0.001), several deliveries (*P* = 0.012), low total household income (*P* = 0.006), cesarean delivery (*P* = 0.015) and were not satisfied for their infant’s gender (*P* = 0.002).

Table 2 shows the crude and adjusted odds ratios for PPD among mothers. Women who were employed and with low gestational age, food insecurity, lower wealth, cesarean delivery and unintended pregnancy had 3.04 (95% CI: 2.95–3.12, *P* < 0.001), 1.19 (95% CI: 1.13–1.28, *P* = 0.02), 1.10 (95% CI: 1.03–1.20, *P* = 0.04), 1.28 (95% CI: 1.17–1.41, *P* < 0.001) and 2.52 (95% CI: 2.23–2.85, *P* < 0.001), times the odds of experiencing symptoms of PPD, respectively; this association remained even after adjustment for confounding variables. Although, women with several deliveries had a lower risk for PPD before and after adjustment (OR = 0.92, 95% CI: 0.88–0.97, *P* < 0.001). Other factors found to be associated with symptoms experience of PPD was dissatisfaction with the gender that was seen significant only before adjustment. Also, the job of the spouse who works as an employee were significant after adjusting for confounders in the adjusted model (OR = 1.25, 95% CI: 1.16–1.32, *P* < 0.001).

The results of the crude and adjusted logistic regressions for postpartum anxiety among women are presented in Table 3. Women who were employed and with low gestational age, employee spouse’s job, cesarean delivery and unintended pregnancy had 2.14 (95% CI: 2.03–2.22, *P* < 0.001), 1.26 (95% CI: 1.18–1.34, *P* < 0.001), 1.14 (95% CI: 1.05–1.25, *P* = 0.01), 1.12 (95% CI: 1.06–1.20, *P* = 0.04) and 1.72 (95% CI: 1.54–1.86, *P* < 0.001), times the odds of experiencing symptoms of postpartum anxiety, respectively; this association remained even after adjustment for confounding variables. Although, women with several deliveries had a lower risk for postpartum anxiety after adjustment for confounders (OR = 0.82, 95% CI: 0.75–0.89, *P* < 0.001) and women with food insecurity had a higher risk for postpartum anxiety after adjustment for confounders (OR = 1.13, 95% CI: 1.08–1.18, *P* = 0.02). Other factors found to be associated with postpartum anxiety symptoms’ experience were lower wealth and dissatisfaction with the gender that was seen significant only before adjustment.

## Discussion

The aim of the present study was to investigate the association between demographic characteristics and attempting of pregnancy with PPD and postpartum anxiety among women referring to community health centres. The research site was an urban area of Zarand City in Iran. The results of this study revealed that a smaller percentage of pregnancies in the Zarand City have unintentionally occurred. This result seems obvious because women living in urban areas are more prone to use contraceptive methods and normally unintended pregnancy among them is lesser than those living in rural areas (31).

In the first part of this study, we hypothesised that there is a relationship between demographic characteristics and PPD and postpartum anxiety in unintended pregnancies versus the intended one. The results showed that women with pregnancies categorised as depression were more likely to have low gestational age, food insecurity, several deliveries, high total household income, cesarean delivery and were not satisfied with their infant’s gender. Also, women with pregnancies categorised as anxiety were more likely to have low gestational age, food insecurity, unintended pregnancy, several deliveries, low total household income and cesarean delivery and were not satisfied with their infant’s gender.

Then we hypothesised that there is an association between the demographic characteristics and attempting of pregnancy and the risk of PPD. The results showed that employed women with low gestational age, faced with food insecurity, lower wealth, cesarean delivery and unintended pregnancy had significant times the odds of experiencing symptoms of PPD while women with several deliveries had a lower risk for PPD before and after adjustment. Other factors found to be associated for experiencing symptoms of PPD was dissatisfaction with the gender that was seen significant only before adjustment. Also, the job of the spouse who works as an employee was significant after adjusting for confounders in the adjusted model.

Finally, we hypothesised that there is an association between the demographic characteristics and attempting of pregnancy and the risk of anxiety. The results showed that employed women with low gestational age, employee spouse’s job, cesarean delivery and unintended pregnancy had significant times the odds of experiencing symptoms of postpartum anxiety while women with several deliveries had a lower risk for postpartum anxiety after adjustment for confounders. The results revealed that women with food insecurity had a higher risk for postpartum anxiety after adjustment for confounders. Other factors found to be associated with postpartum anxiety symptoms’ experience were lower wealth and dissatisfaction with the gender that was seen significant only before adjustment.

Preterm birth increases the risk of adverse outcomes and recent research has begun to focus on potential adverse outcomes of birth between 37 and 38 weeks gestation, namely early-term births (32). Chang et al. (33) reported that the offspring of prenatally depressed women had lower gestational age in weeks. Henrichs et al. (34) and Nasreen et al. (35) also reported a significant association between prenatal depression and low birth weight related to low gestational age at term. Among the most important causes of depression and anxiety are socioeconomic factors (36). Food insecurity that related to poverty and low-income is an important problem in the developing world and has considerable impacts on nutrition status, as well as mental-physical growth and development (37). Ezzeddin et al. (38) reported that food insecurity is an influential factor to predispose mothers to depression. Previous research has also demonstrated that the pain associated with giving birth may increase the risk of PPD. In this context, it is stated that cesarean pain linked to PPD. The findings of Sadat et al. (39) demonstrated that vaginal delivery leads to better physical and mental health related to depression decrement and quality of life increment.

While offspring gender is among risk factors for PPD (40), some studies conducted in traditional Western societies report no association between offspring gender and PPD (41). The research results of Goyal et al. (42) confirms the results of our study. They stated that depression was significantly higher in fathers of female infants, compare with fathers who had male infants.

The obtained results of our study about the significant relationship between PPD and anxiety and employment are contradictory. The results of some research indicated that employment as social support may play a critical role in both the development and the treatment/resolution of PPD after birth. For this reason, many clinicians encourage mothers of young children who are at risk for depression to consider ways to optimise their employment circumstances and ‘other’ social support (43). However the contribution of social support to maternal mental health may be particularly important for mothers who return to formal employment after giving birth and in the case of high working pressure can lead to depression (44, 45) showed that income plays an effective role in decreasing the depression and anxiety of pregnant women, so that with increasing household income, depression and anxiety decreased. Based on the research of Khodakarami et al. (45), the husband’s job has also been effective for reducing the depression and anxiety of pregnant women, so that the pregnant women’s depression and anxiety are reduced with the job’s stable of their husbands (46). The reason for this result could be that Iranian working mothers face very low maternity leave after delivery, occupational stress and fear of dismissing from employment.

The results of the statistical tests showed that women who had experienced an unintended pregnancy had a higher degree of depression than women with an intended pregnancy. The pregnancy period represents an important turning point in the lifecycle of every woman and also her family that is associated with enormous biological, psychological and social challenges for the mother to be (47). These challenges will be intensified if they occur unintended and unforeseen. Because of this, a high percentage of pregnancies occur unintendedly is one of the health indicators of women anxious and decrement in quality of life in society (48). Study of the behaviors during and after pregnancy in unwanted pregnancies can be considered as a practical and scientific guide for doing interventions to improve the mental health of women. Preventing unintended pregnancies can reduce depression, anxiety and suicide in women and also reducing mother and infant mortality. Facing to unplanned pregnancy and lack of its agreement brings about pregnancy with more fear and anxiety (49). Moshki and Cheravi (50) achieved similar results in the study of depression in unintended and intended pregnancies in the community of pregnant women in Gonabad City. Moshki and Cheravi (50) reported that the scale of depression was significantly higher in unintended pregnancies than the intended ones. They mentioned that unintended pregnancies cause women to be exposed to more stressor factors, therefore they experience higher levels of anxiety and depression (50). The research results of Zanganeh et al. (51) also confirm the results of this research. They stated that unwanted pregnancy affects PPD. According to their research results, women who gave birth with intended pregnancies didn’t experience depression, while most of the women who gave birth with unintended pregnancies were depressed (52). Stressful life events including lower wealth, food insecurity and adverse experiences before pregnancy are connected to PPD and anxiety (53). Maybe for many mothers, the experience of pregnancy and childbirth is often being accompanied by less likely to be depressed and anxious that in the present research we obtained this result.

One of the limitations of this study was the lack of comparison in terms of depression and anxiety among rural women and urban women, other relative limitations of the present study were the lack of investigation of disorders such as domestic violence, addiction or drug abuse. The strength of this study is using the Edinburgh questionnaire, which is a specific inventory for PPD. It is recommended that gynecologists and midwives of the health centres and clinics follow up on the history of mothers with unintended pregnancies for PPD and postpartum anxiety.

## Conclusion

In summary, our study indicates that low gestational age, cesarean delivery and unintended pregnancy are among the considerable risk factors affecting the occurrence of PPD and anxiety among the sample while women with several deliveries had a lower risk for PPD and postpartum anxiety. According to the results of this study, it seems that having more children has an effective role in reducing mothers’ PPD and anxiety. Unlike previous studies, there was a significant relationship between woman employment and depression. This shows the need for more postpartum support for employed women in Iran. The findings of the present research are correlational and therefore, should be interpreted with caution. Certainly, there is a great need for long-term longitudinal studies to survey more factors with a deeper study that may be associated with depression and anxiety among diverse populations of mothers after pregnancy.

## Figures and Tables

**Table 1:** Relationship between demographic characteristics and postpartum depression and anxiety

Demographic information		Depression *N* (%)			Level of anxiety *N* (%)			*P*-value

		Without depression	With depression	*P*-value	Mild	Moderate	Severe	
Gestational age, weeks	< 27	3 (0.7)	2 (0.5)	< 0.001	2 (0.5)	1 (0.2)	2 (0.5)	< 0.001
	28–33	7 (1.8)	3 (0.7)		4 (1.0)	3 (0.7)	2 (0.5)	
	34–36	16 (4.0)	6 (1.5)		15 (3.7)	4 (1.0)	3 (0.7)	
	≥ 37	273 (68.2)	90 (22.6)		254 (63.5)	85 (21.2)	61 (15.2)	
Food status	Food security	48 (12)	39 (9.7)	0.039	32 (8.0)	47 (11.7)	8 (2.0)	
	Mild food insecure	187 (46.7)	74 (18.5)		70 (17.5)	155 (38.7)	37 (9.2)	0.048
	Severely food insecure	38 (9.5)	14 (3.5)		21 (5.2)	16 (4.0)	15 (3.7)	
Education	Uneducated	31 (8.7)	21 (5.2)	0.124	28 (7.1)	18 (5.4)	6 (1.5)	
	Less than the diploma	91 (24.8)	35 (8.8)		98 (24.7)	24 (6.1)	4 (1)	0.130
	Diploma	76 (19)	53 (13.2)		79 (19.9)	43 (10.8)	5 (1.3)	
	University student	62 (15.5)	31 (8.7)		57 (14.4)	27 (8.6)	7 (1.8)	
Occupation	Housewife	131 (32.8)	77 (19.2)	0.402	138 (34.8)	58 (14.6)	11 (2.8)	0.964
	Employed	129 (32.2)	63 (15.8)		124 (31.3)	54 (13.6)	11 (2.8)	
Socio-economic status	Lower wealth	44 (11)	43 (10.8)	0.008	22 (5.6)	54 (13.5)	11 (2.8)	0.006
	Adequate wealth	177 (44.2)	84 (21)		59 (14.9)	165 (41.7)	38 (9.6)	
	Higher wealth	39 (9.8)	13 (3.2)		16 (4.0)	25 (6.2)	9 (2.2)	
Spouse’s job	Self-employment	83 (20.8)	44 (11)	0.060	79 (19.9)	38 (6.6)	10 (2.5)	0.055
	Employee	114 (28.2)	60 (15)		114 (24.8)	52 (13.1)	8 (1.8)	
	Other	63 (15.8)	63 (9)		69 (17.4)	22 (5.6)	5 (1.3)	
Numbers of the deliveries	First delivery	141 (35.2)	63 (15.8)	0.004	130 (32.8)	54 (13.6)	18 (5.4)	0.012
	Several deliveries	119 (29.8)	77 (19. 2)		132 (33.3)	58 (14.6)	4 (10)	
Type of delivery	Vaginal	158 (39.5)	67 (16.8)	0.015	152 (38.4)	59 (14.9)	12 (3)	0.026
	Cesarean	102 (25.5)	73 (18.2)		110 (27.8)	53 (13.4)	10 (5.2)	
Attempting of pregnancy	Intended	143 (35.8)	117 (29.2)	0.009	129 (32.6)	54 (13.6)	14 (3.5)	0.001
	Unintended	57 (14.2)	83 (20.8)		133 (33.6)	62 (6.14)	8 (2)	
Satisfaction with the gender	Yes	120 (30.0)	140 (35.0)	0.002	139 (35.1)	67 (16.9)	10 (2.5)	0.329
	No	64 (16.0)	76 (19.0)		118 (29.5)	51 (12.7)	15 (3.7)	

**Table 2:** The association between the demographic characteristics of mothers and the risk of postpartum depression

Characteristics		OR (95% CI)	*P*-value	Adjusted* OR (95% CI)	*P*-value
Gestational age, weeks	≥ 37	1		1	
	34–36	2.01 (2.00–2.13)	< 0.001	2.09 (1.98–2.21)	< 0.001
	28–33	1.96 (1.89–2.07)	< 0.001	1.98 (1.93–2.08)	< 0.001
	< 27	3.04 (2.95–3.12)	< 0.001	3.00 (2.93–3.10)	< 0.001
Food status	Food security	1		1	
	Not severely food insecure	1.14 (1.09–1.22)	0.023	2.09 (2.05–2.16)	< 0.001
	Severely food insecure	1.12 (1.08–1.26)	0.039	2.08 (2.04–2.13)	< 0.001
Education	Uneducated	1		1	
	Less than the diploma	1.06 (1.00–1.13)	0.226	1.04 (0.92–1.11)	0.591
	Diploma	0.96 (0.89–1.07)	0.541	0.98 (0.92–1.07)	0.674
	University student	1.04 (0.95–1.12)	0.423	1.01 (0.93–1.10)	0.818
Occupation	Housewife	1		1	
	Employed	1.19 (1.13–1.28)	0.022	1.25 (1.16–1.32)	< 0.001
Socio-economic status	Higher wealth	1		1	
	Adequate wealth	1.11 (1.08–1.28)	0.021	1.02 (0.96–1.11)	0.415
	Lower wealth	1.10 (1.03–1.20)	0.042	1.25 (1.13–1.31)	< 0.001
Spouse’s job	Self-employment	1		1	
	Employee	1.01 (0.93–1.08)	0.925	1.31 (1.14–1.50)	< 0.001
	Other	1.02 (0.95–1.09)	0.556	1.07 (0.96–1.19)	0.235
Numbers of the deliveries	First delivery	1		1	
	Several deliveries	0.92 (0.88–0.97)	< 0.001	0.85 (0.78–0.93)	< 0.001
Type of delivery	Vaginal	1		1	
	Cesarean	1.28 (1.17–1.41)	< 0.001	1.31 (1.14–1.50)	< 0.001
Attempting of pregnancy	Intended	1		1	
	Unintended	2.52 (2.23–2.85)	< 0.001	1.65 (1.37–2.00)	< 0.001
Satisfaction with the gender	Yes	1		1	
	No	1.24 (1.16–1.33)	< 0.001	1.10 (0.98–1.23)	0.098

Notes:

* adjusted for sex, age, weight, energy intake and physical activity

**Table 3:** The association between the demographic characteristics of mothers and the risk of postpartum anxiety

Characteristics		OR (95% CI)	*P*-value	Adjusted* OR (95% CI)	*P*-value
Gestational age, weeks	≥ 37	1		1	
	34–36	1.11 (1.00–1.22)	0.041	2.12 (2.03–2.21)	< 0.001
	28–33	1.92 (1.86–2.23)	< 0.001	2.05 (1.96–2.12)	< 0.001
	< 27	2.14 (2.03–2.22)	< 0.001	3.10 (2.99–3.22)	< 0.001
Food status	Food security	1		1	
	Not severely food insecure	1.05 (1.00–1.13)	0.196	1.16 (1.08–1.23)	0.016
	Severely food insecure	1.02 (0.93–1.07)	0.525	1.13 (1.08–1.18)	0.021
Education	Uneducated	1		1	
	Less than the diploma	1.02 (0.90–1.11)	0.697	1.07 (1.00–1.15)	0.195
	Diploma	0.99 (0.92–1.07)	0.855	0.97 (0.89–1.06)	0.613
	University student	1.05 (0.96–1.12)	0.481	1.04 (0.96–1.12)	0.442
Occupation	Housewife	1		1	
	Employed	1.26 (1.18–1.34)	< 0.001	1.24 (1.15–1.31)	< 0.001
Socio-economic status	Higher wealth	1		1	
	Adequate wealth	1.11 (1.08–1.28)	0.031	1.01 (0.94–1.08)	0.899
	Lower wealth	0.97 (0.90–1.05)	0.127	0.98 (0.92–1.06)	0.695
Spouse’s job	Self-employment	1		1	
	Employee	1.14 (1.05–1.25)	0.012	1.22 (1.13–1.35)	< 0.001
	Other	1.06 (0.98–1.15)	0.213	1.13 (1.08–1.22)	0.021
Numbers of the deliveries	First delivery	1		1	
	Several deliveries	0.95 (0.86–1.04)	0.111	0.82 (0.75–0.89)	< 0.001
Type of delivery	Vaginal	1		1	
	Cesarean	1.12 (1.06–1.20)	0.044	1.15 (1.07–1.24)	0.016
Attempting of pregnancy	Intended	1		1	
	Unintended	1.72 (1.54–1.86)	<0.001	2.21 (2.13–2.39)	< 0.001
Satisfaction with the gender	Yes	1		1	
	No	1.12 (1.06–1.16)	0.038	1.04 (0.98–1.10)	0.515

Notes:

* adjusted for sex, age, weight, energy intake, and physical activity

## References

[1] Rashidi Fakari F, Simbar M, Saei Ghare Naz M, Rashidi Fakari F (2018). Factors related to empowering iranian women’s fertility behaviors: a systematic review. J Obstet Gynecol Cancer Res.

[2] Van Vo T, Hoa TKD, Hoang TD (2017). Postpartum depressive symptoms and associated factors in married women: a cross-sectional study in Danang City, Vietnam. Front Public Health.

[3] Affonso DD, De AK, Horowitz JA, Mayberry LJ (2000). An international study exploring levels of postpartum depressive symptomatology. J Psychosom Res.

[4] Klainin P, Arthur DG (2009). Postpartum depression in Asian cultures: a literature review. Int J Nurs Stud.

[5] Azad R, Fahmi R, Shrestha S, Joshi H, Hasan M, Khan ANS (2019). Prevalence and risk factors of postpartum depression within one year after birth in urban slums of Dhaka, Bangladesh. PloS One.

[6] Sinesi A, Maxwell M, O’Carroll R, Cheyne H (2019). Anxiety scales used in pregnancy: systematic review. BJ Psych Open.

[7] Martini J, Petzoldt J, Einsle F, Beesdo-Baum K, Höfler M, Wittchen H-U (2015). Risk factors and course patterns of anxiety and depressive disorders during pregnancy and after delivery: a prospective-longitudinal study. J Affect Disord.

[8] Shahhosseini Z, Pourasghar M, Khalilian A, Salehi F (2015). A review of the effects of anxiety during pregnancy on children’s health. Mater Sociomed.

[9] Rafiee B, Akbarzade M, Asadi N, Zare NJJoh (2013). Comparison of attachment and relaxation training effects on anxiety in third trimester and postpartum depression among primipara women. HAYAT.

[10] Verreault N, Da Costa D, Marchand A, Ireland K, Dritsa M, Khalifé S (2014). Rates and risk factors associated with depressive symptoms during pregnancy and with postpartum onset. J Psychosom Obstet Gynaecol.

[11] Ganjouei TA, Karim Zadeh Z, Faramarzi Gohar A, Hosseini-Zijoud SS, Hosseini-Zijoud SM (2015). Unwanted pregnancy and related causes in pregnant women in Kerman, 2013. Pajouhan Scientific Journal.

[12] Shahbazin S, Gholami A (2015). Prevalence of unintended pregnancy and its related factors in Kermanshah, Kangavar city (West Iran). J Community H Research.

[13] Mosalanejad L (2013). Effective factor on delivery and on choosing its type in Iran: a phenomenological study. Intl J Public H Science.

[14] Wigert H, Johansson R, Berg M, Hellström AL (2006). Mothers’ experiences of having their newborn child in a neonatal intensive care unit. Scand J Caring Sci.

[15] Zaheri F, Ranaie F, Karimeh R, Shahoi R (2015). Unwanted pregnancy and associated factors among pregnant women who referred to Sanandaj health centers in 2011. IJOGI.

[16] Araban M, Bahrami N, Karimian Z, Khasaeiyan S (2013). Comparison of unintended and intended pregnancy outcomes. ANM.

[17] Eliason S, Baiden F, Yankey BA, Awusabo–Asare K (2014). Determinants of unintended pregnancies in rural Ghana. BMC (BMC Pregnancy Childbirth).

[18] Jarahi L, Zavar A, Neamat Shahi M (2014). Evaluation of the frequency of unwanted pregnancy and its related factors in the pregnant women of Sarakhs city. IJOGI.

[19] Parhizkar A, Sahsavari S (2016). Comparison of infants’ growth indicators in wanted and unwanted pregnancy at health centers of Sanandaj in 2014–2015. IJOGI.

[20] Khajehpour M, Simbar M, Jannesari S, Ramezani-Tehrani F, Majd HA (2013). Health status of women with intended and unintended pregnancies. Public Health.

[21] Nazarpour S, Tehrani FR, Simbar M, Azizi F (2015). Thyroid dysfunction and pregnancy outcomes. Iran J Reprod Med.

[22] Dahl J, Wilson KG, Nilsson AJBt (2004). Acceptance and commitment therapy and the treatment of persons at risk for long-term disability resulting from stress and pain symptoms: a preliminary randomized trial. Behavior Therapy.

[23] Ericksen J, Condon J, Bilszta J, Brooks J, Milgrom J, Hayes B (2005). Recognition and management of perinatal depression in general practice: a survey of GPs and postnatal women. Aust Fam Physician.

[24] Hou Y, Hu P, Zhang Y, Lu Q, Wang D, Yin L (2014). Cognitive behavioral therapy in combination with systemic family therapy improves mild to moderate postpartum depression. Braz J Psychiatry.

[25] Eliason S, Baiden F, Yankey BA, Awusabo–Asare KJBp, childbirth (2014). Determinants of unintended pregnancies in rural Ghana. BMC Pregnancy Childbirth.

[26] Ahluwalia IB, Johnson C, Rogers M, Melvin C, Group PW (1999). Pregnancy Risk Assessment Monitoring System (PRAMS): unintended pregnancy among women having a live birth. J Womens Health Gend Based Med.

[27] Rafiei M, Nord M, Sadeghizadeh A, Entezari MH (2009). Assessing the internal validity of a household survey-based food security measure adapted for use in Iran. Nutr J.

[28] Petrozzi A, Gagliardi L (2013). Anxious and depressive components of Edinburgh Postnatal Depression Scale in maternal postpartum psychological problems1. J Perinat Med.

[29] Sharma V, Sommerdyk C (2013). Are antidepressants effective in the treatment of postpartum depression? A systematic review. Prim Care Companion CNS Disord.

[30] Hosmer DW, Lemeshow S (2000). Applied logistic regression.

[31] Ali A, Ali SA, Aziz Ali S, Khuwaja NS (2016). Determinants of unintended pregnancy among women of reproductive age in developing countries: a narrative review. JMRH.

[32] Crump C, Sundquist K, Winkleby MA, Sundquist J (2013). Early-term birth (37–38 weeks) and mortality in young adulthood. Epidemiology.

[33] Chang HY, Keyes KM, Lee K-S, Choi IA, Kim SJ, Kim KW (2014). Prenatal maternal depression is associated with low birth weight through shorter gestational age in term infants in Korea. Early Hum Dev.

[34] Henrichs J, Schenk J, Roza S, Van den Berg M, Schmidt H, Steegers E (2010). Maternal psychological distress and fetal growth trajectories: the generation R study. Psychol Med.

[35] Nasreen HE, Kabir ZN, Forsell Y, Edhborg M (2010). Low birth weight in offspring of women with depressive and anxiety symptoms during pregnancy: results from a population based study in Bangladesh. BMC (BMC Public Health).

[36] Hahn-Holbrook J, Cornwell-Hinrichs T, Anaya I (2018). Economic and health predictors of national postpartum depression prevalence: a systematic review, meta-analysis, and meta-regression of 291 studies from 56 countries. Front Psychiatry.

[37] Weaver LJ, Hadley C (2009). Moving beyond hunger and nutrition: a systematic review of the evidence linking food insecurity and mental health in developing countries. Ecol Food Nutr.

[38] Ezzeddin N, Jahanihashemi H, Zavoshy R, Noroozi M (2018). The prevalence of postpartum depression and its association with food insecurity among mothers referring to community health centers. Iran J Psychiatry.

[39] Sadat Z, Taebi M, Saberi F, Kalarhoudi MA (2013). The relationship between mode of delivery and postpartum physical and mental health related quality of life. Iran J Nurs Midwifery Res.

[40] Goldbort J (2006). Transcultural analysis of postpartum depression. Am J Matern Child Nurs.

[41] Robertson E, Grace S, Wallington T, Stewart DE (2004). Antenatal risk factors for postpartum depression: a synthesis of recent literature. Gen Hosp Psychiatry.

[42] Goyal K, Purbiya P, Lal SN, Kaur J, Anthwal P, Puliyel JM (2017). Correlation of infant gender with postpartum maternal and paternal depression and exclusive breastfeeding rates. Breastfeeding Med.

[43] Gjerdingen D, McGovern P, Attanasio L, Johnson PJ, Kozhimannil KB (2014). Maternal depressive symptoms, employment, and social support. J Am Board Fam Med.

[44] Milgrom J, Gemmill AW, Bilszta JL, Hayes B, Barnett B, Brooks J (2008). Antenatal risk factors for postnatal depression: a large prospective study. J Affect Disord.

[45] Khodakarami B, Golalizadeh Bibalan F, Soltani F, Soltanian A, Mohagheghi H (2017). Impact of a counseling program on depression, anxiety, stress, and spiritual intelligence in pregnant women. JMRH.

[46] Khorramirad A, Lotfi MM, Bidgoli AS (2010). Prevalence of postpartum depression and related factors in Qom. Pejouhandeh.

[47] Podvornik N, Velikonja VG, Praper P (2015). Depression and anxiety in women during pregnancy in Slovenia. Zdr Varst.

[48] Biaggi A, Conroy S, Pawlby S, Pariante CM (2016). Identifying the women at risk of antenatal anxiety and depression: a systematic review. J Affect Disord.

[49] Anokye R, Acheampong E, Budu-Ainooson A, Obeng EI, Akwasi AG (2018). Prevalence of postpartum depression and interventions utilized for its management. Ann Gen Psychiatry.

[50] Moshki M, Cheravi K (2016). Relationships among depression during pregnancy, social support and health locus of control among Iranian pregnant women. IJSP.

[51] Zanganeh M, Shams N, Alizadeh K, Rezaei M, Pormher S (2009). Postpartum depression and its relationship with unwanted pregnancy and baby gender. JKUMS.

[52] Zanganeh M, Shams AN, Kaamrvamanesh M, Rezaei M, Pormher S (2009). Postpartum depression and its relationship with unwanted pregnancy and baby gender. Journal Kurdistan Univ Med Sci.

[53] Kettunen P, Koistinen E, Hintikka J (2016). The connections of pregnancy-, delivery-, and infant-related risk factors and negative life events on postpartum depression and their role in first and recurrent depression. Depress Res Treat.

